# Chemical Characteristics and Source-Specific Health Risks of the Volatile Organic Compounds in Urban Nanjing, China

**DOI:** 10.3390/toxics10120722

**Published:** 2022-11-24

**Authors:** Jingyun Wang, Hao Yue, Shijie Cui, Yunjiang Zhang, Haiwei Li, Junfeng Wang, Xinlei Ge

**Affiliations:** Jiangsu Key Laboratory of Atmospheric Environment Monitoring and Pollution Control, Collaborative Innovation Center of Atmospheric Environment and Equipment Technology, School of Environmental Science and Engineering, Nanjing University of Information Science and Technology, Nanjing 210044, China

**Keywords:** aromatics, positive matrix factorization, health risks, ozone formation potential, biogenic source

## Abstract

This work comprehensively investigated the constituents, sources, and associated health risks of ambient volatile organic compounds (VOCs) sampled during the autumn of 2020 in urban Nanjing, a megacity in the densely populated Yangtze River Delta region in China. The total VOC (TVOC, sum of 108 species) concentration was determined to be 29.04 ± 14.89 ppb, and it was consisted of alkanes (36.9%), oxygenated VOCs (19.9%), halogens (19.1%), aromatics (9.9%), alkenes (8.9%), alkynes (4.9%), and others (0.4%). The mean TVOC/NO_x_ (ppbC/ppbv) ratio was only 3.32, indicating the ozone control is overall VOC-limited. In terms of the ozone formation potential (OFP), however, the largest contributor became aromatics (41.9%), followed by alkenes (27.6%), and alkanes (16.9%); aromatics were also the dominant species in secondary organic aerosol (SOA) formation, indicative of the critical importance of aromatics reduction to the coordinated control of ozone and fine particulate matter (PM_2.5_). Mass ratios of ethylbenzene/xylene (E/X), isopentane/n-−pentane (I/N), and toluene/benzene (T/B) ratios all pointed to the significant influence of traffic on VOCs. Positive matrix factorization (PMF) revealed five sources showing that traffic was the largest contributor (29.2%), particularly in the morning. A biogenic source, however, became the most important source in the afternoon (31.3%). The calculated noncarcinogenic risk (NCR) and lifetime carcinogenic risk (LCR) of the VOCs were low, but four species, acrolein, benzene, 1,2-dichloroethane, and 1,2-dibromoethane, were found to possess risks exceeding the thresholds. Furthermore, we conducted a multilinear regression to apportion the health risks to the PMF-resolved sources. Results show that the biogenic source instead of traffic became the most prominent contributor to the TVOC NCR and its contribution in the afternoon even outpaced the sum of all other sources. In summary, our analysis reveals the priority of controls of aromatics and traffic/industrial emissions to the efficient coreduction of O_3_ and PM_2.5_; our analysis also underscores that biogenic emissions should be paid special attention if considering the direct health risks of VOCs.

## 1. Introduction

Volatile organic compounds (VOC) are an important group of compounds that can greatly affect air quality and human health. Although on a global scale, VOC emissions are dominated by natural/biogenic sources. In an urban region, anthropogenic sources (such as fossil fuel combustion, biomass burning, chemical processing, solvent use, etc.) are often more important [1]. High concentrations of total VOCs (TVOCs) have been observed in several megacities in China, such as Beijing (89.29 ppb) [2], Shijiazhuang (121.4 ppb) [3], Shanghai (94.14 ppb) [4], Guangzhou (129.2 ppb) [5], and Chengdu (108.45 ppb) [6]. Stringent VOC emission control is currently a priority of air pollution remediation measures imposed by the Chinese government, largely since VOCs are important precursors of both secondary fine particulate matter (PM_2.5_) and ozone—the two major pollutants that lead to haze and photochemical smog, respectively. Especially, in general, VOCs can participate in complex photochemical reactions and production of free radicals, which promote the circulation of NO_x_ and lead to the generation of tropospheric ozone [7]. Ozone itself is also a greenhouse gas, so a rise in ground-level ozone concentration contributes to global warming [8]. At the same time, since plants mainly absorb tropospheric ozone through leaf stomata, the increase in ozone concentration can oxidize chlorophyll cells and damage plant photosynthesis, affecting plant health [9,10]. Ozone can cause adverse effects on the respiratory system, inducing breathing difficulties, airway inflammation [8], asthma, emphysema, and chronic bronchitis [11,12]. Among the VOCs, alkanes, alkenes, and aromatics are typically abundant VOC types in ambient air in China [13,14,15,16], yet the contributions of different VOCs to ozone or secondary organic aerosol (SOA) formations do not necessarily correspond to their relative abundances but are dependent upon their reactivities. For example, aromatics are often not the dominant VOC type in terms of mass concentration but might be dominant in ozone formation; similarly, aromatics, like toluene, benzene, ethylbenzene, etc., can play an important role in SOA formation as well, despite their low mass loadings [17,18,19,20].

Some VOCs are a great concern directly to human health. For instance, certain aldehydes, ketones, and amines can cause serious irritation to human respiratory mucosa, eyes, and skin; long-term exposure to these VOCs can cause cancer and deformities, and even endanger lives [21,22,23,24]. To be specific, formaldehyde is an important allergen in ambient air, which can aggravate breathing difficulties and induce bronchial hypersensitivity, regardless of whether there is a long-term smoking habit [25]. Health effects of acetaldehyde include irritation of the skin, eyes, and mucous membranes, causing vomiting, headache, and cancer risk [26,27]. Ketones, such as acetone, can cause acute poisoning of the human body, fatigue, nausea, headache, and eye irritation; repeated contacts with the skin can lead to degreasing and dermatitis [28]. Another chemical, 2-butanone, can cause neurological symptoms (headaches, fatigue, and feelings of intoxication) in humans, irritating the eyes, nose, and mucous membranes; renal congestion, mild renal necrosis, and organ weight gain have been observed in laboratory animal studies [29]. Amines, such as dimethylnitrosamine, dimethylnitrosamine, and acrylamide are possible strong carcinogens to humans [30]. Moreover, many VOCs have odorous properties, such as ethylamine, trimethylamine, formaldehyde, styrene, and mercaptans [31,32]. The odor of some VOCs is unpleasant and has a negative effect on people’s quality of life, in particular, the residents of nearby emission sources [33]. The discomfort of the human body, attributed to odorous gases, can lead to inefficient labor and damage to health, making people irritable, and unfocused, and causing lowered judgment and reduced memory, etc. [34,35].

This work aims to elucidate the composition, sources, and health risks associated with ambient VOCs in a representative densely populated city (Nanjing, in the Yangtze River Delta region, China). Importantly, we quantify the source-specific health risks of VOCs by linking the source profiles resolved from positive matrix factorization (PMF) with their estimated carcinogenic and noncarcinogenic risks. Our findings are valuable for effective VOCs control and the reduction of their health hazards in the future.

## 2. Experimental Methods

### 2.1. Sampling Site, Instrumentation, and Chemical Analysis

**Figure 1:**
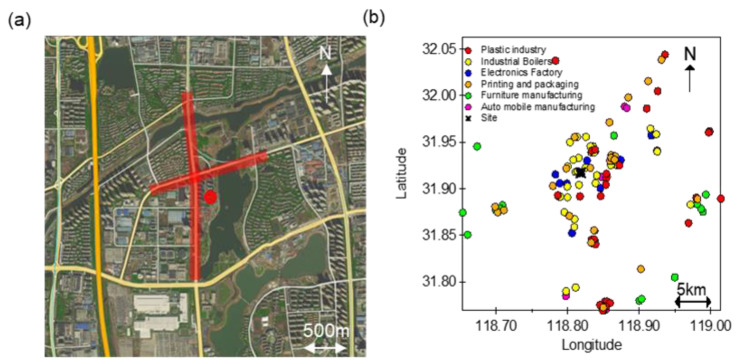
(**a**) Location of the sampling site (red dot) and its surroundings (red lines denote two arterial roads); (**b**) Spatial distribution of industrial plants (colored by different types) around the site (black star).

The sampling site was located in the Jiangning District of urban Nanjing, China (118.818161° E, 31.917282° N) (Figure 1a), and VOC samples were acquired in the open space on the rooftop of a 10-story building (~35 m above the ground). The site was close to the arterial roads in the west and north (~250 m away, with high traffic flows), and was surrounded by residential buildings, office buildings, industrial plants, and schools. This site thus represents a typical urban environment with VOCs released from multiple anthropogenic sources such as traffic, industrial, and residential activities. To better understand the impact of possible industrial emissions, we marked the locations of nearby factories in Figure 1b. These factories include electronics, machinery manufacturing and maintenance, plastic processing, printing, and painting, and the majority of them were located in the east/northeast of the site.

The sampling period was from 11 October to 12 November 2020. The VOCs were sampled twice a day at ~9:00 a.m. and ~3:00 p.m. respectively, each lasting for one hour, by using SUMMA canisters (6.0 L, Entech Instruments Inc., Simi Vally, CA, USA), and a total of 60 valid samples were obtained. Before sampling, the canisters were cleaned by a canister cleaner (3100D, Entech Instruments Inc., Simi Vally, CA, USA) at least 3 times and then were pumped to vacuum (<50 mtorr) before use. The sampled VOCs were enriched by a preconcentrator (7200, Entech Instruments Inc., Simi Vally, CA, USA) using the ECTD (Extended Cold Trap Dehydration) technology to remove interferences from water and carbon dioxide. The VOCs were then analyzed by a gas chromatography mass spectrometer (GC-MS, 7890B/5977A, Agilent Technologies, Inc., Boulder, CO, USA) [36]. In total, 108 compounds were measured, including 30 alkanes, 34 halogens, 17 aromatics, 13 alkenes, 12 oxygenated volatile organic compounds (OVOCs), 1 alkyne, and 1 other compound.

The quality control and quality assurance of GC-MS mainly include the multipoint calibration (using PMAS and TO-15 mixed standard gases, Sigma-Aldrich, St. Louis, MO, USA), single-point calibration, and mass spectrometric tuning (using 4−bromofluorobenzene (BFB) 1 μL (50 ng)). The procedure of the multipoint calibration was as follows: the standard gas was diluted to a 10 nmol/mol working standard by using a dynamic dilution apparatus (4600D, Entech Instruments Inc., Simi Vally, CA, USA); a univariate linear regression was used to create the calibration curve by inputting results from six standards (20 mL, 50 mL, 100 mL, 200 mL, 400 mL, and 600 mL), and the correlation coefficients (r^2^) of the regressions for all compounds were assured to be >0.99, with a response factor relative standard deviation (RF RSD) of 6.1~26.8%. The detection limits of 108 VOCs ranged from 0.002 ppb to 0.050 ppb.

Concentrations of other pollutants including O_3_ (Model 49i, Thermo Fisher, Waltham, MA, USA), NO_x_ (Model 42i, Thermo Fisher, Waltham, MA, USA ), CO (Model 48i, Thermo Fisher, Waltham, MA, USA), SO_2_ (Model 43i, Thermo Fisher, Waltham, MA, USA), and PM_2.5_ (XHQ500C, XianHe Co. Ltd., Shijiazhuang, China), and the meteorological data (XHPM200E, XianHe Co. Ltd., Shijiazhuang, China) including temperature, relative humidity, wind speed, wind direction, and PM2.5 were all obtained from the nearby national environmental monitoring station (~50 m away).

### 2.2. Data Analysis

#### 2.2.1. Source Apportionment

PMF is a powerful and widely used tool to resolve distinct sources of pollutants based on measured data without prior information regarding the source profiles [37,38,39]. This study used the US EPA (Environmental Protection Agency) PMF 5.0 software kit [40,41,42,43] for source apportionment of VOCs. The PMF algorithm decomposes the measured data matrix into the product of two matrices (factor profiles and time series of the factors’ concentrations) plus a residual matrix. One advantage of PMF is that it carefully weighs the measurement uncertainties, making its solution robust, meaningful, and reliable. In this work, the uncertainty of a measured VOC was calculated by Equation (1):

(1) $$\mathrm{Unc}=\sqrt{{\left(\mathrm{Error}\;\mathrm{Fraction}\times C\right)}^{2}+{\left(0.5\times \mathrm{MDL}\right)}^{2}}$$

where Error Fraction is chosen as 0.1 here, C is the concentration of a VOC (in ppb), and MDL is the method detection limit of that VOC. Measured data below the MDL were replaced by 1/2 MDL and corresponding uncertainties were set to 5/6 MDL. Some missing data were substituted by the geometric mean of its neighborhood measured values with an uncertainty of 4 times the uncertainty of the measured values. The data were then classified into three categories according to their signal-to-noise (uncertainty) (S/N) ratios. The data points with S/N ratio < 0.2 were classified as “bad” and were discarded; data with S/N ratios of 0.2–2 were treated as “weak” and were down weighted by increasing the corresponding uncertainties by three times; those with S/N ratios > 2 were regarded as “strong” and were directly used in PMF analysis.

In addition, we also took into account the species which are recognized as specific source markers. After the pretreatment, 35 VOCs were chosen, including 11 alkanes, 4 alkenes, 1 alkyne, 5 halogens, 8 aromatics, and 6 OVOCs. The PMF solutions were considerably evaluated by exploring different numbers of factors, rotational ambiguity, and bootstrapping (100 runs) for an estimation of the uncertainty of the solution, following the standard protocol in the EPA PMF 5.0 operation manual. Finally, we selected the 5-factor solution as the best result (see details in Section 3.3.2).

#### 2.2.2. Calculation of Ozone Formation Potential (OFP) and Secondary Organic Aerosol Formation Potential (SOAFP)

VOC reactivity is critical to the formation of ozone and SOA. A number of studies focused on the mechanisms of ozone formation [44,45] and proposed different approaches to quantify the OFPs of VOCs [46,47,48]. A widely used and simplified method is described by Equation (2) [49]:

(2) $${\mathrm{OFP}}_{i}=\left[{\mathrm{VOC}}_{i}\right]\times {\mathrm{MIR}}_{i}$$

where OFP_i_ (in μg/m^3^) refers to the OFP of VOC_i_; VOC_i_ (in μg/m^3^) refers to the measured concentration of VOC_i_, and MIR_i_ is the value of maximum increment reactivity of VOC_i_. In this study, the VOCs’ MIR values were adopted from those documented in Carter [50], and are available for most VOCs (93 out of 108).

Similar to OFP, SOAFP quantifies the ability of a VOC to generate SOA [51,52,53], which is shown in the following equation (details in Hui et al. [54]):

(3) $${\mathrm{SOAFP}}_{i}=\left[{\mathrm{VOC}}_{i}\right]\times {\mathrm{SOAP}}_{i}$$

where SOAFP_i_ (μg/m^3^) is the SOAFP of VOC_i_, and SOAP_i_ refers to the coefficient of VOC_i_ to form SOA. The SOAP_i_ values used here were from Derwent et al. [55]. The values are only available for 31 VOCs. Although the SOA formation depends on various environmental factors, the use of SOAP_i_ allows reasonable estimations of contributions of individual precursors to SOA formation and demonstrates the relative importance of these precursors [56].

#### 2.2.3. Health Risk Assessment

We performed a quantitative health risk assessment on the VOCs by using the method recommended by the US EPA and the Exposure Factor Handbook of Chinese Population (US EPA, 1989; Ministry of Ecology and Environment of China, 2013), which have been widely used previously [57,58,59]. There are two health risk indicators: Lifetime carcinogenic risk (LCR) and noncarcinogenic risk (NCR). The calculation equations for a certain VOC are listed below:

(4) $$\mathrm{EC}=\frac{\left(\mathrm{CA}\times \mathrm{ET}\times \mathrm{EF}\times \mathrm{ED}\right)}{\mathrm{AT}}$$

(5) LCR=IUR×EC

(6) $$\mathrm{NCR}=\frac{\mathrm{EC}}{\left(\mathrm{RfC}\times 1000\right)}$$

(7) HI=∑HQi

Here, EC is the exposure concentration (in μg/m^3^); CA is the ambient (measured) concentration (in μg/m^3^); ET is the exposure time (hours/day); EF is the exposure frequency (days/year); ED is the years of exposure (years), and AT is the average time (hours). In this study, EF is 365 d/year from the US EPA Integrated Risk Information System (IRIS), ET is 3.7 h/day, ED is 74.8 years, and AT is therefore 74.8 × 365 × 24 h, all from the Exposure Factor Handbook of Chinese Population (Adult) based on Ministry of Ecology and Environment of China. IUR is the inhalation unit risk (in m^3^/μg), RfC is the reference concentration (in mg/m^3^). The IUR and RfC values of different species were obtained from the risk assessment information system (RAIS) developed by the University of Tennessee. The values are compiled in Table S1 in the Supplementary Materials. We were able to calculate LCR for 16 VOCs and NCR for 39 VOCs.

## 3. Results and Discussion

### 3.1. Overview of Air Pollutants and Meteorological Conditions

**Figure 2:**
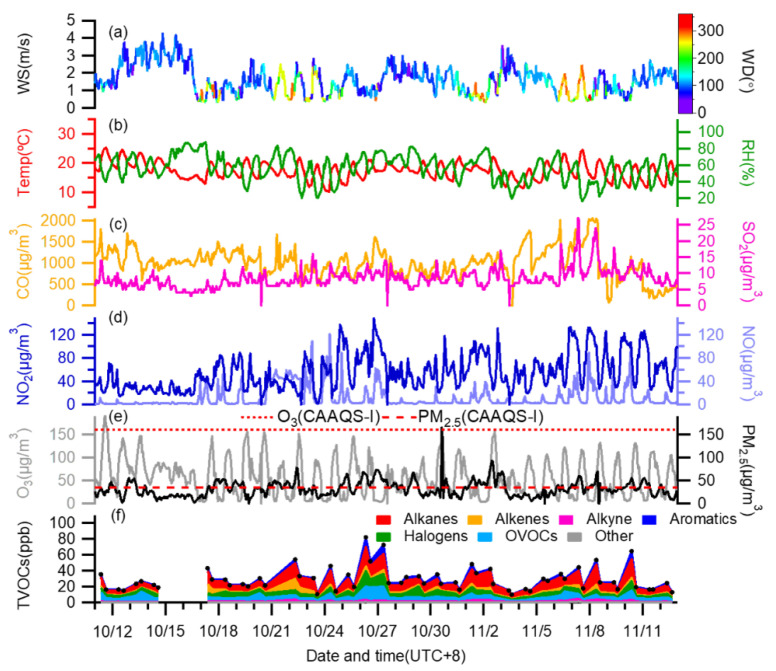
Time series of (**a**) wind speed (WS) colored by wind direction (WD), (**b**) relative humidity (RH) and temperature, concentrations of (**c**) CO, SO_2_, (**d**) NO_2_, NO, (**e**) O_3_, PM_2.5_, and (**f**) the total VOCs (TVOCs) during the whole campaign.

The time series of CO, NO_x_ (NO and NO_2_), PM_2.5_, SO_2_, O_3_, different groups of VOCs, and the meteorological parameters during the campaign are shown in Figure 2. The wind was prevailing from the east and southeast (Figure S1). The average wind speed was relatively low at 1.60 ± 0.81 m/s (±one standard deviation, same hereafter). The RH level was moderate with a mean of 56.46 ± 15.62%, and the mean temperature was 17.38 ± 2.89 °C. For PM_2.5_, over half of the days met with the Grade I of Chinese ambient air quality standard (CAAQS-I) (<35 μg/m^3^), and the average mass loading was 32.21 ± 16.62 μg/m^3^. For the diurnal pattern, PM_2.5_ concentration was relatively low during the 10:00 a.m.–6:00 p.m. range (except the peak at 9:00 a.m. likely owing to traffic emissions), corresponding to atmospheric conditions that favor diffusion and evaporation (higher temperature, lower RH, stronger wind as shown in Figure S2) [60,61]. O_3_ was the opposite, relatively high during the daytime (8:00 a.m.–6:00 p.m.) (Figure S2). NO_x_ and CO peaked during the morning rush hours (6:00–9:00 a.m.) clearly as a result of enhanced traffic activities, and then decreased, likely due to their consumptions for O_3_ production and atmospheric dilution.

### 3.2. Chemical Characteristics of VOCs

#### 3.2.1. Mass Concentration and Composition

**Figure 3:**
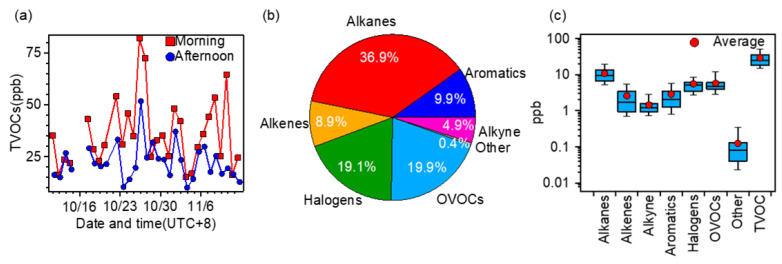
(**a**) Temporal variations of TVOC concentrations in the morning and afternoon, (**b**) mass contributions of different VOCs to TVOC, and (**c**) box plots of concentrations of different VOCs (the whiskers above and below the boxes mark the 90% and 10% percentiles, respectively; the upper and lower edges of the boxes represent the 75% and 25% percentiles, respectively; and the lines and red dots inside the boxes denote the median and mean values, respectively).

During the campaign, the TVOC concentrations (sum of 108 species) ranged from 9.96 to 81.97 ppb (Figure 2), with an average of 29.04 ± 14.89 ppb. We further presented the results in the morning and afternoon, respectively, in Figure 3a. The highest TVOC concentration (81.97 ppb) appeared on the morning of 26 October, which was approximately eight times the lowest one (9.97 ppb) on the afternoon of 3 November. In general, the VOC level in the morning was significantly higher than that in the afternoon (on average 35.84 ppb vs. 22.24 ppb). This is in fact a common feature of ambient VOCs with a few possibilities: One reason is due to enhanced VOC emissions in the morning from sources such as traffic, etc.; another reason is that low temperature, low wind speed and low planetary boundary layer (PBL) height in the morning exacerbate the accumulation of emitted VOCs. Finally, but importantly, strong solar radiation in the afternoon can lead to strong photochemical loss of VOCs, therefore decreasing its ambient level [62].

As shown in Figure 3b, on a mass average, the TVOC was consisted of 36.93% alkanes, 19.89% OVOCs, 19.06% halogens, 9.85% aromatics, and other minor species (8.90% alkenes, 4.95% alkyne, etc.). Compared with a previous study in Nanjing [19], alkanes remain the most abundant VOCs, followed by OVOCs and halogens. In detail, the average concentrations of alkanes, alkenes, alkyne, aromatics, halogens, OVOCs, and other compounds were 10.72 ± 5.27 ppb, 2.58 ± 2.55 ppb, 1.43 ± 0.74 ppb, 2.86 ± 2.43 ppb, 5.53 ± 3.14 ppb, 5.77 ± 3.58 ppb, and 0.12 ± 0.16 ppb, respectively (Figure 3c). The concentrations of alkanes, alkenes, and aromatics were generally lower than earlier results in Nanjing [42,43,63]. Compared to a previous study conducted in a similar period in Nanjing [64], the TVOC concentration is about half. Compared with results in other major cities in China (selected studies that cover the five economically developed city clusters in China, as shown in Table S2), the TVOC level here was also at a lower end, close to those in Guangzhou, Wuhan and Liangyungang, but much lower than those in Shanghai, Beijing, and Chengdu. Note the differences might be largely due to coverage of VOCs, sampling times, and environments (urban or suburban). Nevertheless, this work covered a relatively wide range of VOCs, therefore a low VOCs level indicates an improvement in VOC pollution in the Yangtze River Delta region. Regarding the different VOC types, in most cases (Table S2), alkanes were the most abundant one, with significant contributions from aromatics and alkenes, similar across different cities.

#### 3.2.2. Relationship with NO_x_

**Figure 4:**
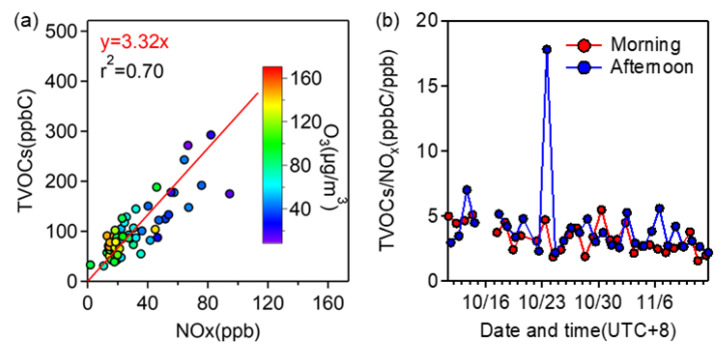
(**a**) Scatter plot of concentrations of TVOC versus NO_x_, and (**b**) temporal variations of VOCs/NO_x_ (ppbC/ppbv) ratios in morning and afternoon samples, respectively.

As is well known, O_3_ formation responds to VOCs and NO_x_ levels nonlinearly, and the VOCs/NO_x_ ratio is critical to O_3_ control [65,66]. As already shown in Figure S2, NO_x_ concentration peaked in the morning due to elevated traffic activities, gradually went down, and reached a minimum at ~2:00 p.m.; on the other hand, the diurnal trend of O_3_ was nearly opposite to that of NO_x_. Next, we showed the scatter plot of TVOCs versus NO_x_ in Figure 4a. VOCs and NO_x_ concentrations correlated tightly with an *r*^2^ of 0.70, and high O_3_ concentrations were typically accompanied by both low NO_x_ and VOCs concentrations, indicating clearly that the co-consumption of NO_x_ and VOCs led to O_3_ generation. The slope of TVOC versus NO_x_ was only 3.32. Such a low VOCs/NO_x_ (ppbC/ppbv) ratio (much less than 8 [66]) means that O_3_ control is VOC-limited rather than NO_x_-limited. This result agrees with another study in Nanjing [67]. Thus, the reduction of VOC emissions should be more effective than NO_x_ reduction in this region to O_3_ abatement.

Furthermore, we calculated the VOCs/NO_x_ ratios in the morning and afternoon, respectively, in Figure 4b. As expected, the average value in the morning (3.31) was lower than in the afternoon (average 4.11); but both values were still far below eight, meaning that the consumption of NO_x_ does not alter the O_3_ control regime. There was only one value larger than eight, in the afternoon of 23 October, which was accompanied by extremely low NO_x_ (average 1.84 ppb), relatively high VOCs (32.84 ppbC), and O_3_ concentrations (average 88.00 μg/m^3^).

### 3.3. Contributions of VOCs to Ozone and SOA Formations

#### 3.3.1. Contributions to OFP

**Figure 5:**
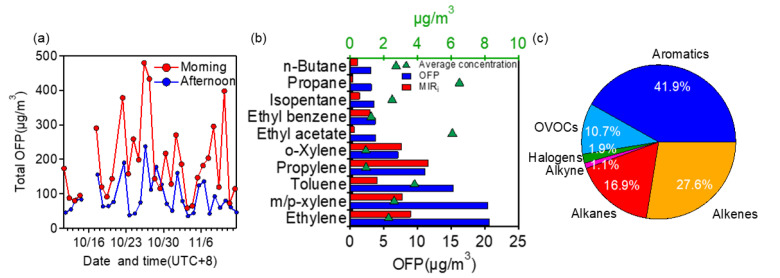
(**a**) TVOC OFP values for samples in the morning and afternoon, (**b**) top 10 species of OFP (as well as their MIR values and average concentrations), and (**c**) relative contributions of different VOCs to the OFP.

The average total OFP of all samples was calculated to be 140.27 ± 3.81 μg/m^3^. Compared with results from other major cities in China, it is close to those in Changzhi (145.80 μg/m^3^) [68], and Zhengzhou (183.00 μg/m^3^) [57], much lower than those in Shanghai (249.70 μg/m^3^) [69], Chuzhou (273.25 μg/m^3^) [51], Guangzhou (800.00 μg/m^3^) [70], and much higher than those in Huaian (97.35 μg/m^3^) [51] and Chengdu (74.50 μg/m^3^) [71]. Figure 5a further shows the time series of calculated OFP during morning and afternoon respectively. The largest OFP of 479.59 μg/m^3^ was on the morning of 26 October, and the lowest one was 35.30 μg/m^3^ on the afternoon of 3 November. On average, OFP in the morning (190.19 ± 111.92 μg/m^3^) was more than two times that in the afternoon (90.36 ± 50.59 μg/m^3^); as a comparison, the average TVOC concentration in the morning was 1.6 times that in the afternoon.

Figure 5b illustrates the top ten VOCs in terms of OFP. Ethylene, m/p xylene, and toluene had large OFP values of 20.67 μg/m^3^, 20.46 μg/m^3^, and 15.33 μg/m^3^, respectively. Note their mass contributions to TVOC were only 2.6%, 3.1%, and 4.5%, but contributions to total OFP were 14.7%, 14.6%, and 10.9%, respectively. The three species are in fact frequently reported to be the ones with the largest OFP in many other regions of China. Other compounds in Figure 5b were propylene, o-xylene, ethyl acetate, ethylbenzene, isopentane, propane, and n-butane with OFP values of 11.12 μg/m^3^, 7.17 μg/m^3^, 3.83 μg/m^3^ 3.80 μg/m^3^, 3.64 μg/m^3^, 3.18 μg/m^3^, and 3.15 μg/m^3^, and contributions of 7.9%, 5.1%, 2.7%, 2.7%, 2.6%, 2.3%, and 2.2%, respectively. Specifically, propane was the single most abundant species (7.61%) in terms of mass, yet its contribution to OFP was much lower (2.27%) due to its small MIR value. Overall, these ten VOCs accounted for 34.78% of the TVOC concentration but occupied 65.84% of the TVOC OFP. Regarding VOC categories, as shown in Figure 5c, aromatics accounted for the largest portion (41.9%) of OFP, followed by alkenes (27.6%) and alkanes (16.9%). This is contrasting to their mass contributions (Figure 3c), where aromatics, alkenes, and alkanes accounted for 9.9%, 8.9%, and 36.9% of TVOC, respectively. These findings collectively suggest the priority of VOC control to ozone formation should focus on the top ten species in Figure 5b as well as the aromatics.

#### 3.3.2. Contributions to SOAFP

**Figure 6:**
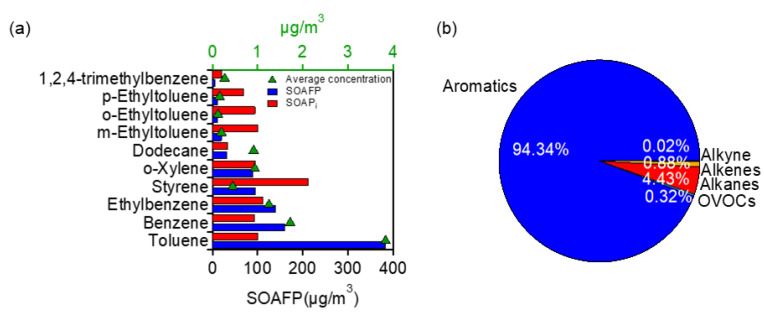
(**a**) Top 10 species of SOAFP (as well as their SOAP values and average concentrations), and (**b**) relative contributions of different VOCs to the SOAFP.

Similar to OFP, we also calculated the SOAFP of different VOCs. The top ten species of SOAFP are presented in Figure 6a, namely, toluene (383.33 ± 358.16 μg/m^3^), benzene (159.87 ± 107.00 μg/m^3^), ethylbenzene (139.59 ± 141.78 μg/m^3^), styrene (94.95 ± 113.12 μg/m^3^), o-xylene (89.61 ± 96.04 μg/m^3^), dodecane (31.47 ± 36.77 μg/m^3^), m-ethyltoluene (19.86 ± 17.07 μg/m^3^), o-ethyltoluene (11.56 ± 10.13 μg/m^3^), p-ethyltoluene (11.12 ± 12.04 μg/m^3^), and 1,2,4-trimethylbenzene (5.54 ± 4.95 μg/m^3^). The average total SOAFP was 976.96 ± 69.91 μg/m^3^, higher than those in Beijing (767.40 μg/m^3^) and Xuchang (860.00 μg/m^3^) [72], much lower than that in Wuhan (1661–4542 μg/m^3^) [54], and close to that in Chengdu (>930.00 μg/m^3^) [73]. Similar to that of OFP, the mean SOAFP value was also notably higher in the morning than in the afternoon (1333.55 ± 898.63 vs. 620.37 ± 393.26 μg/m^3^).

The largest SOAFP contributor was toluene (39.24%), though its mass contribution to TVOC was only 4.49%. The mass concentration of the top ten species occupied only 11.53% of TVOC mass, but their contribution to SOAFP was 96.36%. It should be noted that the available SOAP_i_ values are quite limited (only 31 compounds) compared to available MIR values (93 compounds). Among the 31 species, most of them are aromatic compounds, thereby the contribution to total SOAFP was overwhelmingly dominated by aromatics (94.34%), while contributions from other VOC types were very minor (4.43% from alkenes, 0.88% from alkanes, and 0.32% from OVOCs). Nevertheless, despite this limitation and uncertainty, this result points out the effectiveness of the control of aromatics to PM_2.5_ reduction (by reducing SOA formation). Together with the results in Figure 6b, our analysis reveals that aromatics are the common key species to the coordinated control of both PM_2.5_ and O_3_.

### 3.4. Sources of VOCs

#### 3.4.1. Diagnostic Ratios

The mass ratios of some specific VOCs can act as indicators of specific sources of VOCs. These diagnostic ratios include ethylbenzene/xylene (E/X), isopentane/n-pentane (I/N), and toluene/benzene (T/B) ratios. The E/X ratio can indicate photochemical age, as ethylbenzene and xylene have the same origin, yet the reaction rate constant of xylene against hydroxyl radical is three times that of ethylbenzene [74]. Therefore, a high E/X ratio would signify a high aging degree as the photochemical loss of xylene is much faster than that of ethylbenzene under the same conditions. Here, the E/X ratio was only 0.58 ± 0.17 (ppb/ppb). Compared with the value of 1.28 ± 0.36 on polluted days and 1.20 ± 0.21 on clean days in Wuhan [54], our result demonstrates the observed VOCs were relatively fresh and thus closely linked with local emissions.

Since reaction rate constants of isopentane and n-pentane against the hydroxyl radical are close [75], and they are often coemitted, the I/N ratios determined directly from different sources are therefore used to infer these sources for ambient VOCs [76], such as natural gas (0.82–0.89), liquefied petroleum gas (2.2–3.8), automobile emissions (1.5–3.0), and liquid gasoline and fuel volatilization (1.8–4.6) [76,77]. The I/N ratio was measured to be 1.50 ± 0.30 (ppb/ppb) in this study, indicating that VOCs in this site were heavily influenced by automobile (traffic) emissions. The T/B ratio can be used to probe the influence of traffic emissions also; generally, a T/B ratio of less than two means a significant influence from traffic, and a larger value would imply less traffic and more influence from other sources. For instance, a previous study shows that the T/B ratio is about 10 if the air is in the vicinity of strong industrial emissions [78]. The T/B ratio was only 2.02 ± 1.49 (ppb/ppb) in this study, suggesting again the large impact from traffic, similar to an earlier study that reports a T/B ratio of 1.5~3.0 in the VOCs affected by urban traffic [79]. Schauer et al. [80] report a T/B ratio of 1.79 for emissions directly from a gasoline vehicle bench test (and a tolune/ethylbenzene ratio of 5.10, compared to that of 3.67 ± 1.41 in this study). In summary, both I/N and T/B ratios demonstrates that the ambient VOCs in the site were greatly affected by traffic emissions.

#### 3.4.2. Source Apportionment

**Figure 7:**
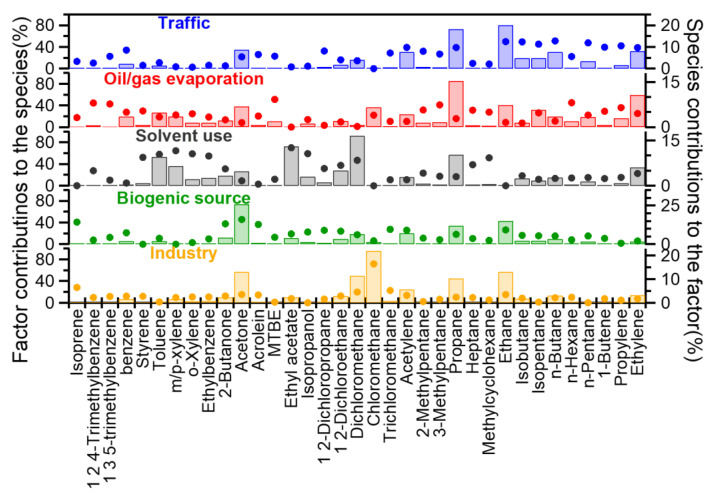
Mass profiles of the five PMF-resolved factors (solid dots refer to the factor contributions to each species; bars represent the mass contributions of each species to the factors).

The PMF analysis separated five VOCs sources, and their profiles are illustrated in Figure 7. The first factor contained relatively high fractions of ethane, ethylene, propane, propylene, acetylene, etc. Acetylene is often used as a tracer of automobile exhaust, C3-C5 alkenes, and alkanes are also abundant in traffic emissions as a result of the incomplete combustion of gasoline/diesel [81,82]. Therefore, this factor is clearly associated with traffic. The second factor was significantly rich in 2-methylpentane, 3-methylpentane, methyl tert-butyl ether, and n-hexane, with contributions of 31.61%, 41.05%, 51.19%, and 45.15% to their total concentrations, respectively. The chemicals 2-methylpentane and 3-methylpentane are closely related to gasoline evaporation [83]. Methyl tert-butyl ether is a known gasoline additive, working as an antiknock additive, and is often regarded as a marker of gasoline-related emissions [39]. Other species from oil/gasoline, including isopentane, n-pentane [84], styrene, and trimethylbenzene [85], were present in this factor as well. The factor is thus denoted as oil/gas evaporation.

Major species in the third factor were benzene, isopropyl alcohol, and ethyl acetate, and numerous researchers have shown that paints and solvents can emit large amounts of these aromatics [86,87,88,89,90]. Moreover, isopropyl alcohol and ethyl acetate are common organic solvents [91], while contributions of this factor to these two species (69.94% and 59.09%) were much higher than in other factors, thus the third factor was very likely from solvent use. Contributions of isoprene, acetone, acrolein, some aldehydes, and ketones from the fourth factor were particularly high. Since isoprene is a well-known tracer of biogenic emissions, and acetone can be emitted from plants too [92,93], this factor was indicative of a biogenic (natural) source. For the last factor, the most plentiful compound was chloromethane (contributing 71.65% to the species), which occupied 21.92% mass of the factor. Meanwhile, contributions from this factor to dichloromethane and trichloromethane were also significant, reaching 19.80% and 22.60%, respectively. These compounds are commonly found in industrial solvents and raw materials [94]. In light of the number of plants (such as plastic processing) located near the sampling site, this factor was denoted as industry.

**Figure 8:**
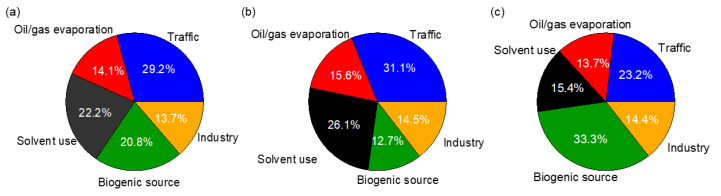
Average mass contributions of different sources to TVOC for (**a**) all samples, (**b**) morning, and (**c**) afternoon samples.

As shown in Figure 8, the average mass contributions of traffic, oil/gas evaporation, solvent use, biogenic source, and industry were 29.2%, 14.1%, 22.2%, 20.8%, and 13.7%, respectively. Traffic and solvent use appeared to be the top two VOCs sources, which can be explained by the nearby busy traffic activities as well as densely distributed plastic processing/printing/packaging plants. VOCs from the other two sources, oil/gas evaporation, and industry, were actually relevant with traffic and industrial activities too. In addition to the anthropogenic sources, natural VOC emissions should not be ignored too. Compared to Wuhan in 2021, the contribution of solvent use (22.2% vs. 11.6%) is significantly high, whereas those of oil and gas evaporation (14.1% vs. 14.2%) and traffic (29.2% vs. 22.5%) are at similar levels; Compared with that in Xinxiang in 2021, traffic contribution is relatively high (29.2% vs. 14.0%), the industry contribution is lower (13.7% vs. 30.0%), and that of solvent use is similar (22.2% vs. 25.0%). Compared to a previous study in Nanjing in 2018, contributions from traffic (29.2% vs. 23.0%), solvent use (22.2% vs. 12.0%), and oil and gas evaporation (14.1% vs. 10.0%) were relatively high, and that of industry (13.7% vs. 30.0%) was low [63,95,96]. This result suggests the necessity of the reinforcement of stringent control of industrial emissions (such as the usage of new green solvents and new technology) in addition to traffic emission control (such as the usage of electronic vehicles).

The relative contributions of different sources in the morning and afternoon samples are shown in Figure 8b,c. It is found that the roles of traffic and solvent use were more significant in the morning than in the afternoon (31.1% vs. 23.2% for the traffic, 26.1% vs. 15.4% for the solvent use), while the biogenic source become the largest contributor in the afternoon (33.3%) from the smallest one in the morning (12.7%).

### 3.5. Health Risks of VOCs

#### 3.5.1. Calculations of LCR and NCR

**Figure 9:**
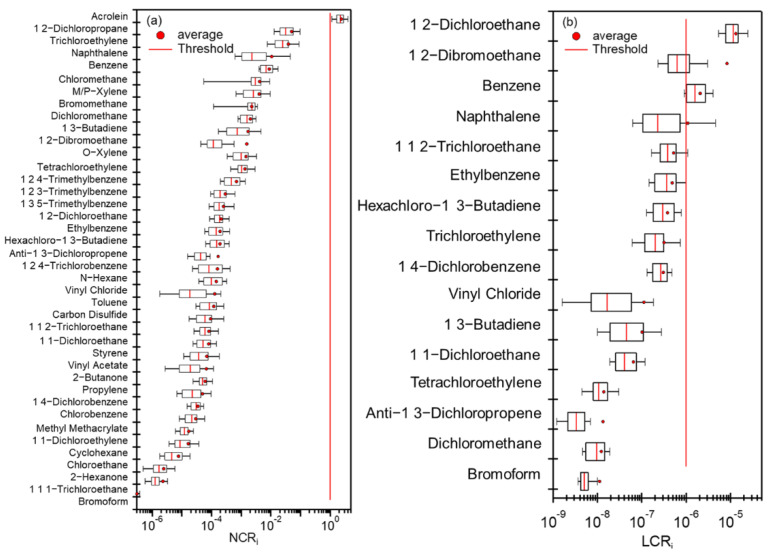
Calculated values of (**a**) noncarcinogenic risk (NCR) and (**b**) lifetime carcinogenic risk (LCR) for different VOCs (symbols of the boxes are the same as those described in Figure 3).

Both LCR and NCR values were calculated for the measured VOCs. The recommended safety thresholds of LCR and NCR are 1 × 10^−6^ and 1 for adults, respectively [57]. As shown in Figure 9, based on the campaign average concentrations, only one compound (acrolein, 2.36), exceeded the NCR threshold, and only two compounds (benzene and 1,2-dichloroethane, 2.07 × 10^−6^ and 13.15 × 10^−6^) exceeded the LCR threshold. LCR values of 1,2-dibromoethane, naphthalene, and 1,1,2-Trichloroethane for a few samples were above the threshold. As shown in Table S2, compared with previous results in other cities such as Langfang [97], Beijing [98], and Zhengzhou [57], the average NCR of VOCs (sum of all calculated NCR divided by the number of calculated VOCs) in this study (2.69 × 10^−5^) was a little higher than those in Langfang (2.54 × 10^−5^) and Beijing(1.58 × 10^−5^), but lower than that in Zhengzhou (5.28 × 10^−5^), while the average LCR value (2.49) was lower than those determined in these cities, except in Langfang (5.17). In particular, acrolein was found to have a high NCR (1.60~4.90) in these cities as well due to its very strict RfC value.

Although our calculation results demonstrate that the overall health risks of VOCs were low, it should be noted that for a large portion of VOCs measured here, the IUR or RfC values are lacking. Therefore, their health risks are not calculated, and thus are unknown. Health risks of some certain species, including acrolein, 1,2-dichloroethane, 1,2-dibromoethane, benzene, naphthalene, and 1,1,2-Trichloroethane should be paid attention and targeted reduction of these species is needed.

#### 3.5.2. Source-Specific Health Risks

**Figure 10:**
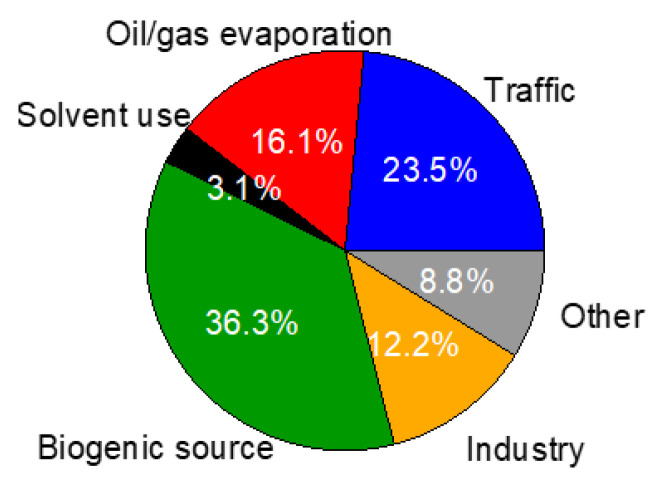
Relative contributions of the PMF-resolved sources to the TVOC NCR.

In order to link the health risks of VOCs with their specific emission sources, we performed a multilinear regression (MLR) between the calculated LCR or NCR and the PMF-resolved factors. This PMF-MLR method worked poorly on TVOC LCR (*r*^2^ of 0.14 between reconstructed and calculated values) but quite well on TVOC NCR (*r*^2^ of 0.67 and can explain ~91.2% of total NCR). The TVOC NCR can then be reasonably separated into contributions of the five sources, e.g., 23.5% from traffic, 16.1% from oil/gas evaporation, 3.1% from solvent use, 36.3% from biogenic source, 12.2% from industry and 8.8% from an unidentified source (denoted as other) (Figure 10). Notably, biogenic source outweighed traffic, becoming the largest contributor to the TVOC NCR.

**Figure 11:**
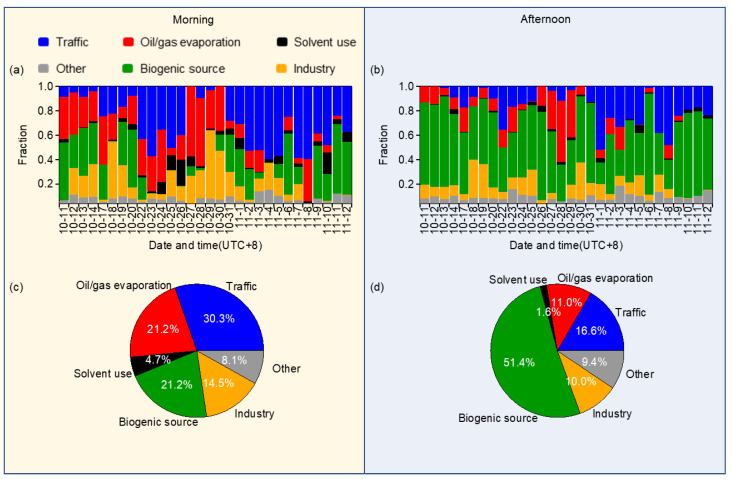
Relative contributions of different sources to the TVOC NCR in all morning samples (**a**) and the average (**c**), and in all afternoon samples (**b**) and the average (**d**).

Figure 11 shows the source contributions to NCR in the morning and afternoon samples, respectively. It can be seen that the contributions varied greatly on different days, indicating the complexity of the health risks of VOCs. On average, the NCR of morning samples was mainly from traffic (30.3%), oil/gas evaporation (21.2%), and biogenic source (21.2%). The contributions of afternoon samples were quite different and it was dominated by biogenic emissions (51.4%), with much fewer contributions from traffic (16.6%) and oil/gas evaporation (11.1%). The main reason may be because of the reduced traffic activities and emissions from oil/gas evaporation in the afternoon. On the other hand, the enhanced biogenic emissions due to increased temperatures and strong solar radiations might elevate the emission ratios of biogenic VOCs from plants, etc. [99]. It is worth to mention that the morning/afternoon difference in the contribution from biogenic sources to TVOC NCR (51.4% vs. 21.2%) was much larger than its morning/afternoon difference of mass contribution (33.3% vs. 12.7%), highlighting the importance of biogenic emissions to the health risks of VOCs, in particular in the afternoon.

**Figure 12:**
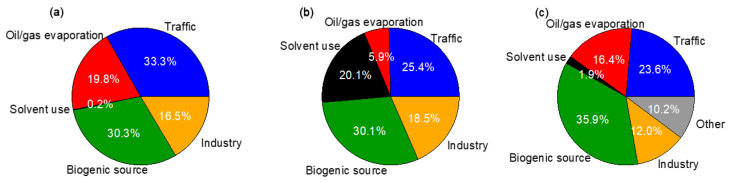
Relative contributions of the PMF-resolved sources to (**a**) LCR of benzene, (**b**) LCR of 1,2-dichloroethane, and (**c**) NCR of acrolein.

In addition, we performed an MLR analysis on the compounds that exceeded the LCR or NCR limits, including benzene, 1,2-dichloroethane, 1,2-dibromoethane, and acrolein (shown in Figure S3). The regression was not satisfied with 1,2-dibromoethane, with an *r*^2^ of only 0.12 between its reconstructed and calculated LCR values. Therefore it was not further discussed. The regressions of the other three compounds were good (*r*^2^ of 0.89, 0.94, and 0.79, respectively). The relative contributions of the five sources to the health risks of these three species are thus presented in Figure 12. The LCR of benzene was mainly contributed to by traffic (33.3%) and biogenic source (30.3%), while the LCR of 1,2-dichloroethane was dominated by biogenic source (30.1%), traffic (25.4%), and solvent use (20.1%). Unlike benzene and 1,2-dichloroethane, the MLR analysis of acrolein resolved an unexplained portion of ~10%; aside from this unknown factor, the top two contributors were biogenic source (35.9%) and traffic (23.6%).

Similarly, the source contributions to LCR and NCR of the aforementioned three species in morning and afternoon samples are shown in Figure S4. The contribution of biogenic source for all three compounds was remarkably higher in the afternoon than in the morning (44.4% vs. 16.2% for LCR of benzene, 44.6% vs. 15.7% for LCR of 1,2-dichloroethane, 50.8% vs. 21.1% for NCR of acrolein). For the morning samples, contributions from traffic (33.9%) and oil/gas evaporation (23.9%) were remarkably high, again underscoring the importance of vehicle-related activities to the LCR of benzene, while solvent use should also be noted for the NCR of 1,2-dichloroethane (27.0%). This result highlights the necessity of targeted control of anthropogenic sources including traffic, oil/gas evaporation, and solvent use to the reduction of health risks of specific VOCs, while control of natural biogenic emissions should also be considered.

## 4. Conclusions

In this study, ambient concentrations of 108 VOCs were determined in urban Nanjing during the autumn of 2020. The mean TVOC concentration was 29.04 ± 14.89 ppb, which was relatively low compared with measurement results in other cities. The average concentration was much higher in the morning than in the afternoon (35.84 ppb vs. 22.24 ppb). Alkanes (36.9%), OVOCs (19.9%), and halogens (19.1%) were the three major VOCs types. The VOCs/NO_x_ (ppbC/ppbv) ratio was on average 3.31 in the morning and 4.11 in the afternoon, demonstrating that ozone control is VOC-limited throughout the day. In contrast, aromatics became the most important VOC group in OFP (41.9%), as well as in SOAFP (94.3%), strongly suggesting that a preference for aromatics control can benefit both ozone and PM_2.5_ reductions.

Diagnostic ratios of E/X, I/N, and T/B all point to the large influence of traffic on VOCs in this site. Further PMF analysis did separate five sources with traffic as the largest contributor (29.2%); solvent use (22.2%), biogenic source (20.8%), oil/gas evaporation (14.1%), and industry (13.7%) were the other four sources. Traffic and solvent use, however, were less important, and biogenic source instead became the largest contributor (33.3%) in the afternoon. Moreover, we calculated the LCR and NCR of measured VOCs and found that the overall health risks were low, except for a few compounds including acrolein, benzene, 1,2-dichloroethane, and 1,2-dibromoethane. The PMF-MLR analysis successfully apportioned the TVOC NCR to individual sources. It is interesting to find that a biogenic source rather than traffic became the most important source to the TVOCs’ NCR and its contribution to the afternoon samples dominated over the sum of all other sources. In summary, our findings reveal the importance of controlling aromatics as well as traffic/industrial emissions to the coordinated reduction of PM_2.5_ and O_3_. In addition, we want to highlight that biogenic emissions should be paid attention to in the future when considering the direct health risks of VOCs.

## Data Availability

The data presented in this article are available on request from the corresponding authors.

## Supplementary Materials

The following supporting information can be downloaded at: https://www.mdpi.com/article/10.3390/toxics10120722/s1, Figure S1: Wind rose plot during the sampling period; Figure S2: Diurnal patterns of the meteorological parameters and major air pollutants; Figure S3: Scatter plots of calculated LCR/NCR values versus reconstructed values from PMF-MLR analysis; Figure S4: Relative contributions of different sources to the LCR/NCR of compounds exceeding thresholds; Table S1: The available parameters for NCR and LCR assessment of VOCs and the results of this work, with selected results from other cities in China [57,97,98]; Table S2: Selected studies of VOCs measurements in the five economically developed regions in China [13,14,15,16,41,54,73,88,93,100,101,102,103,104].
