# Supplementary material for: Chemical Characteristics and Source-Specific Health Risks of the Volatile Organic Compounds in Urban Nanjing, China
Source: Toxics. 2022 Nov 24;10(12):722. doi: 10.3390/toxics10120722 (PMC9783090; doi:10.3390/toxics10120722)
Supplement: Supplementary file 1 [file toxics-10-00722-s001.zip › toxics-2033617-supplementary.pdf]

toxics

MDPI

Supplementary Materials: Chemical Characteristics and Source-Specific Health Risks of the Volatile Organic Compounds in Urban Nanjing, China

Jingyun Wang, Hao Yue, Shijie Cui, Yunjiang Zhang, Haiwei Li, Junfeng Wang, Xinlei Ge

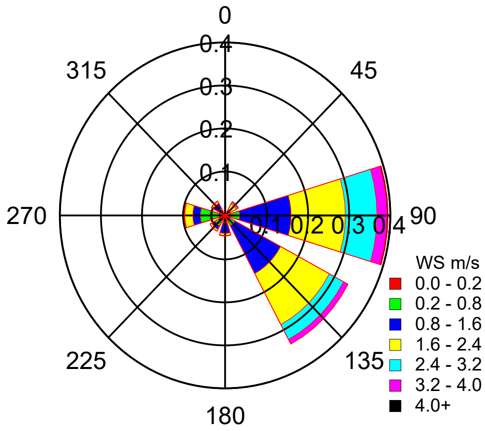

Figure S1. Wind rose plot during the sampling period.

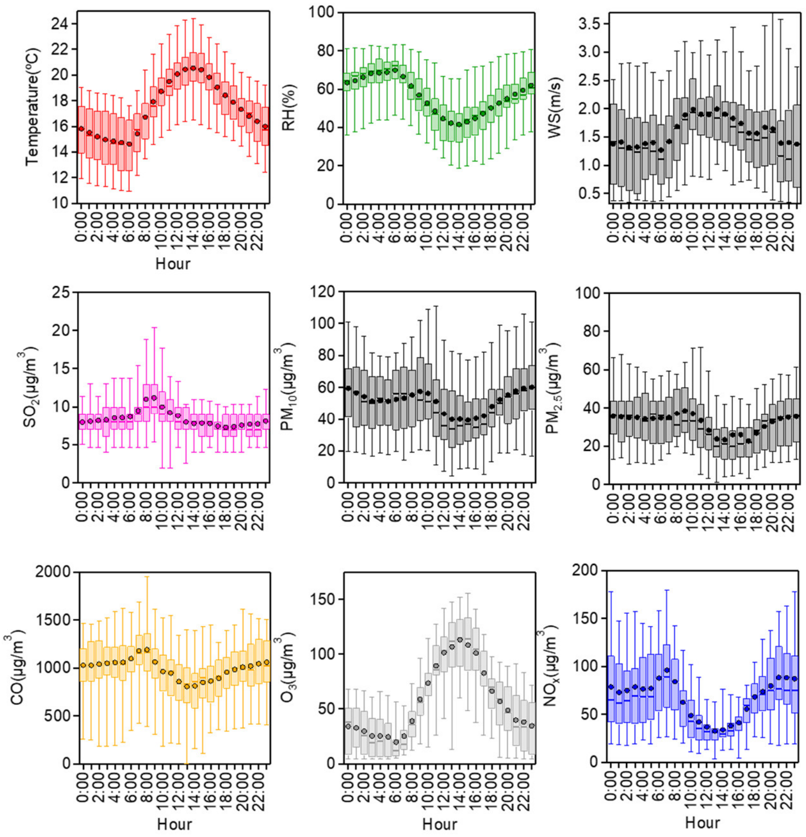

Figure S2. Diurnal patterns of the meteorological parameters and major air pollutants (the whiskers above and below the boxes mark the 90 % and 10 % percentiles, respectively; the upper and lower edges of the boxes represent the 75 % and 25

% percentiles, respectively; and the lines and solid/hollow dots inside the boxes denote the median and mean values, respectively).

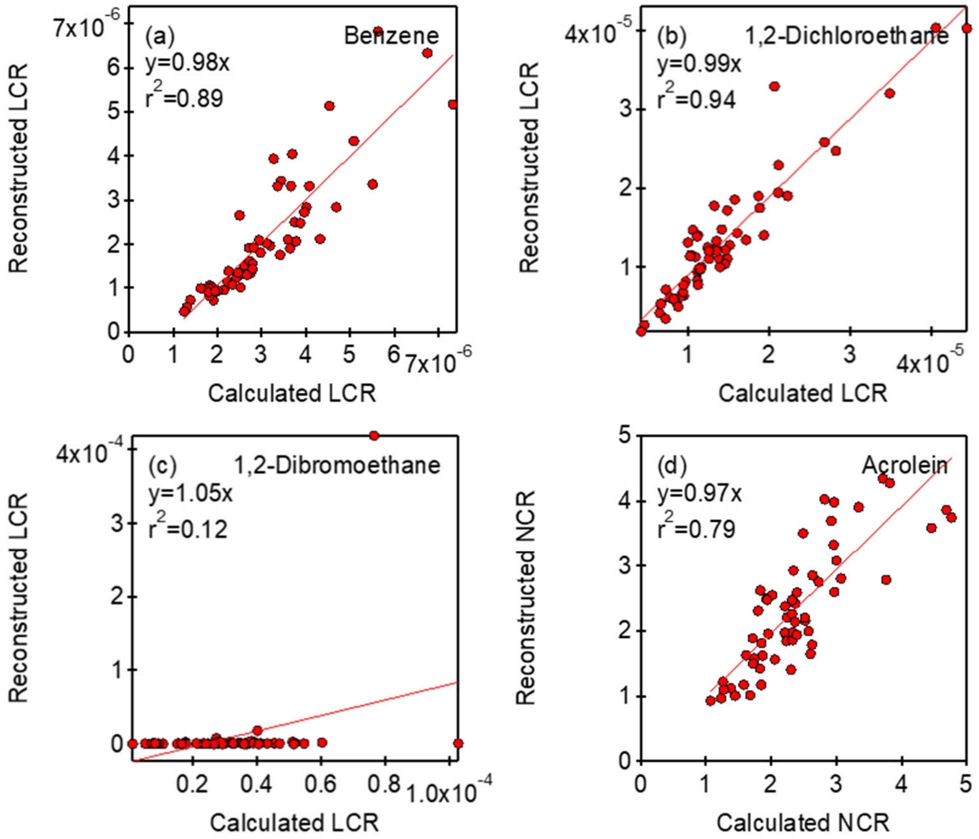

Figure S3. Scatter plots of calculated LCR/NCR values versus reconstructed values from PMF-MLR analysis (see main text), for (a) LCR of benzene, (b) LCR of 1,2-dichloroethane, (c) LCR of 1,2-dibromoethane, and (d) NCR of acrolein.

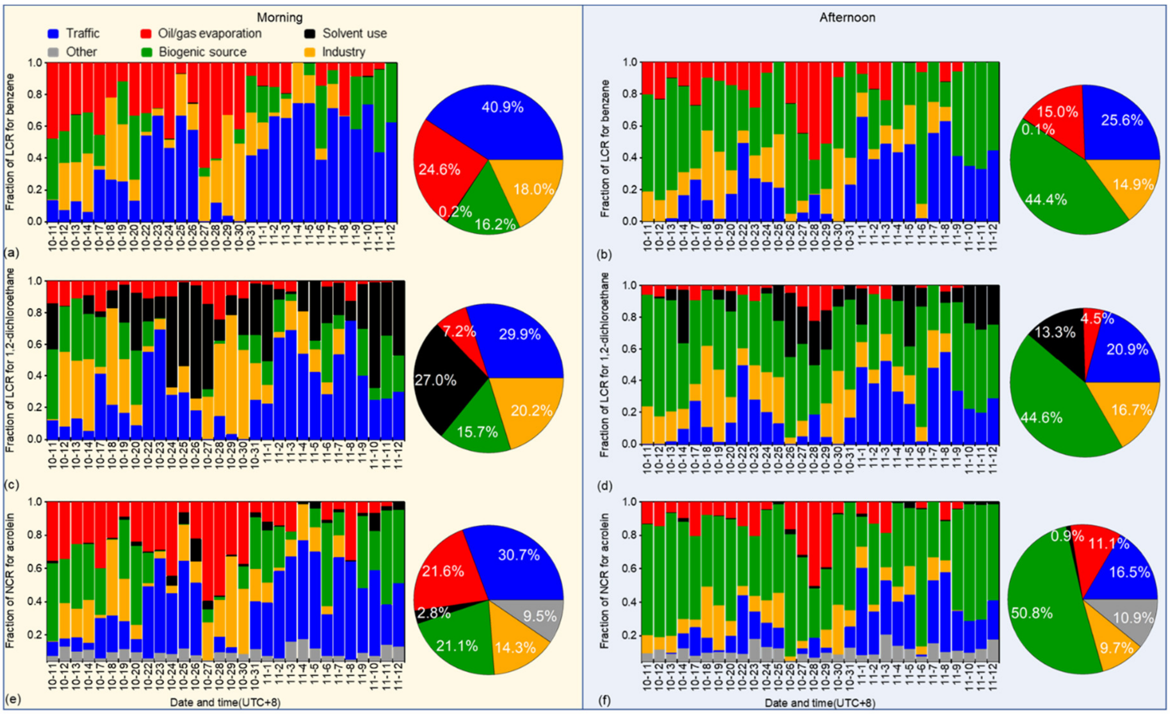

Figure S4. Relative contributions of different sources to the LCR of benzene in morning (a) and afternoon samples (b), to the LCR of 1,2-dichloroethane in morning (c) and afternoon samples (d), and to the NCRof acrolein in morning (e) and afternoon samples (f) (the pie charts are average contributions of corresponding morning or afternoon samples).

Table S1. The available parameters for NCR and LCR assessment of VOCs and the results of this work, with selected results from other cities in China.

VOCs Species	RfC	IUR	Zhengzhou[1]		Langfang[2]		Beijing[3]		This study	
	(mg/m^{-3})	(μg/m^{-3})	NCR	LCR	NCR	LCR	NCR	LCR	NCR	LCR
1,1,1-Trichloroethane	5.00E+00		1.30E-06		4.60E-07		2.40E-07		3.05E-07	
1,1,2-Trichloroethane	4.00E-01	1.60E-05	1.00E-04	6.60E-07		2.30E-07			8.15E-05	5.21E-07
1,1-Dichloroethane	5.00E-01	1.60E-06	6.90E-05	5.50E-08					8.06E-05	6.45E-08
1,1-Dichloroethylene	2.00E-01		1.60E-05						1.64E-05	
1,2,3-Trimethylbenzene	6.00E-02		5.80E-04		5.70E-04				2.88E-04	
1,2,4-Trichlorobenzene	2.00E-01		2.10E-05						1.56E-04	
1,2,4-Trimethylbenzene	6.00E-02		1.70E-03		1.70E-03				6.91E-04	
1,2-Dibromoethane	9.00E-03	6.00E-04	1.40E-03	7.40E-06	2.60E-04	7.10E-07	4.90E-05	2.70E-07	1.54E-03	8.30E-06
1,2-Dichloroethane	2.40E+00	2.60E-05	3.10E-04	1.90E-05					2.11E-04	1.31E-05
1,2-Dichloropropane	4.00E-03		4.30E-02		1.20E-01		8.00E-02		4.98E-02	
1,3,5-Trimethylbenzene	6.00E-02		8.40E-04		7.90E-04				2.52E-04	
1,3-Butadiene	2.00E-03	3.00E-05	1.60E-03	9.40E-08	4.40E-02	2.60E-06	8.10E-03	4.90E-07	1.69E-03	1.01E-07
1,4-Dichlorobenzene	8.00E-01	1.10E-05	1.10E-04	9.30E-07	1.40E-05		5.90E-05		3.46E-05	3.05E-07
1,4-Dioxane	3.00E-02	5.00E-06	5.50E-05	8.20E-09						

2-Butanone	5.00E+00		3.90E-05						6.09E-05	
2-Hexanone	5.00E+00		2.00E-05						2.28E-06	
4-Methyl-2-pentanone	3.00E+00		1.00E-05							
Acrolein	2.00E-05		1.60E+00		4.90E+00				2.36E+00	
Benzene	3.00E-02	7.80E-06	1.80E-02	4.10E-06	3.50E-02	8.30E-06	3.60E-02	8.40E-06	8.84E-03	2.07E-06
Bromoform		1.10E-06		2.70E-08		3.20E-09				1.11E-08
Bromomethane	5.00E-03		1.80E-03		1.30E-03		5.40E-03		2.24E-03	
Carbon disulfide	7.00E-01		1.40E-04						9.29E-05	
Chlorobenzene	1.00E+00		2.90E-05						2.92E-05	
Chloroethane	1.00E+01		1.40E-06						2.43E-06	
Chloroform	9.80E-02	2.30E-05	5.80E-03	1.30E-05		1.30E-05				
Chloromethane	9.00E-02		1.70E-03						4.14E-03	
Cyclohexane	6.00E+00		9.10E-06		1.20E-04		5.20E-05		7.64E-06	
Dichloromethane	6.00E-01	1.00E-08	1.90E-03	1.20E-08					2.03E-03	1.22E-08
Ethylbenzene	1.00E+00	2.50E-06	2.10E-04	5.30E-07	5.40E-04		4.20E-04		1.93E-04	4.82E-07
Hexachloro-1,3-butadiene	9.00E-02	2.20E-05	2.90E-04	5.70E-07					1.92E-04	3.79E-07
Isopropyl benzene	4.00E-01		4.40E-05		9.10E-05		1.50E-05			

m/p-Xylene	1.00E-01		2.00E-03		1.50E-02		1.30E-03		4.04E-03	
Methyl methacrylate	7.00E-01		8.40E-05		2.60E-03	1.60E-08	5.40E-03	3.20E-10	1.70E-05	
MTBE	3.00E+00	2.60E-07	2.80E-05	2.20E-08	2.90E-05					
Naphthalene	3.00E-03	3.40E-05	2.40E-02	2.40E-06					1.06E-02	1.08E-06
n-Hexane	7.00E-01		2.40E-03		4.20E-04		3.40E-04		1.43E-04	
o-Xylene	1.00E-01		7.00E-03		4.90E-03		1.00E-02		1.45E-03	
Propylene	3.00E+00		1.90E-05						4.90E-05	
Styrene	1.00E+00		4.20E-05		2.90E-04		6.50E-05		6.90E-05	
Tetrachloroethylene	4.00E-02	2.60E-07	2.10E-02	2.20E-07	2.50E-02	2.60E-08	2.00E-02	1.90E-07	1.33E-03	1.39E-08
Tetrachloromethane	1.00E-01	6.00E-06	5.90E-03	3.50E-06						
Toluene	5.00E+00		1.50E-04		2.40E-04		1.50E-04		1.18E-04	
trans-1,3-Dichloropropene	2.00E-02	4.00E-06	6.90E-04	5.60E-08					1.66E-04	1.33E-08
Trichloroethylene	2.00E-03	4.10E-06	1.00E-02	8.40E-08	2.00E-02	1.70E-07	2.10E-01	1.70E-06	3.86E-02	3.16E-07
Vinyl acetate	2.00E-01		4.30E-03						6.63E-05	
Vinyl chloride	1.00E-01	8.80E-06	1.30E-04	1.20E-07	3.70E-04	3.30E-07		4.70E-06	1.27E-04	1.12E-07

Table S2. Selected studies of VOCs measurements in the five economically developed regions in China

Sample site	Site Type	Period	Season	VOCs (in ppb)								References
				Alkanes	Alkenes	Alkyne	Aromatics	Halogens	OVOCs	Others	**TVOCs**	
Nanjing	Suburb	2020.10-2020.11	Autumn	10.72	2.58	1.44	2.86	5.69	5.78	0.12	29.04	This study
Beijing	Urban	2019.03	Spring	36.39	8.59	3.91	10.24	0.00	0.00	0.00	59.13	[4]
Beijing	Suburb	2019.04-2019.05	Spring	13.47	2.49	1.61	2.89	3.86	5.29	0.76	30.40	[5]
Beijing	Urban	2016.01-2016.10	Four Seasons	16.19	5.19	3.08	3.39	4.80	11.00	0.31	44.00	[6]

Changsha	Urban	2020.8	Summer	4.50	0.90	0.60	2.80	2.30	6.50	0.00	17.60	[7]
Chengdu	Urban	2018.06-2019.01	Summer, autumn, winter	29.32	6.40	8.53	5.33	3.73	0.00	0.00	53.30	[8]
Chengdu	Urban	2016.05-2017.01	Four Seasons	19.36	4.13	3.17	4.58	4.37	8.98	0.33	44.92	[9]
Chongqing	Suburb	2015.08-2015.09	Summer and autumn	14.63	5.89	4.33	4.82	4.00	6.26	1.24	41.20	[10]
Dongguan	Urban	2020.06-2020.08	Summer	23.90	3.72	0.53	24.96	0.00	0.00	0.00	53.10	[11]
Guangzhou	Suburb	2014.10-2014.11	Autumn	16.66	5.10	3.40	8.84	0.00	0.00	0.00	34.00	[12]
Guangzhou	Urban	2017.09-2017.11	Autumn	20.15	2.40	1.67	12.09	0.00	0.00	0.00	36.30	[13]

Lianyungang	Urban	2018.04-2018.09	Spring and summer	19.31	3.80	0.63	7.91	0.00	0.00	0.00	31.65	[14]
Shanghai	Urban	2017.05	Spring	14.95	2.39	1.20	5.08	5.42	13.66	0.00	42.70	[15]
Wuhan	Urban	2016.09-2017.08	Four Seasons	15.90	4.16	2.43	3.47	3.81	4.94	0.30	34.65	[16]
Wuhan	Urban	2016.10-2016.11	Autumn	13.19	3.83	2.03	3.26	3.59	8.66	0.31	34.87	[17]

References

1. Yu, S.; Su, F.; Yin, S.; Wang, S.; Xu, R.; He, B.; Fan, X.; Yuan, M.; Zhang, R. Characterization of ambient volatile organic compounds, source apportionment, and the ozone–NOx–VOC sensitivities in a heavily polluted megacity of central China: effect of sporting events and. *Atmos. Chem. Phys.* **2021**, *21*, 15239-15257, doi:10.5194/acp-21-15239-2021.
2. Yang, Y.; Ji, D.; Sun, J.; Wang, Y.; Yao, D.; Zhao, S.; Yu, X.; Zeng, L.; Zhang, R.; Zhang, H.; et al. Ambient volatile organic compounds in a suburban site between Beijing and Tianjin: Concentration levels, source apportionment and health risk assessment. *Sci. Total Environ.* **2019**, *695*, 133889, doi:10.1016/j.scitotenv.2019.133889.
3. Gu, Y.; Li, Q.; Wei, D.; Gao, L.; Tan, L.; Su, G.; Liu, G.; Liu, W.; Li, C.; Wang, Q. Emission characteristics of 99 NMVOCs in different seasonal days and the relationship with air quality parameters in Beijing, China. *Ecotoxicol. Environ. Saf.* **2019**, *169*, 797-806, doi:10.1016/j.ecoenv.2018.11.091.
4. Zhang, L.; Wang, X.; Li, H.; Cheng, N.; Zhang, Y.; Zhang, K.; Li, L. Variations in Levels and Sources of Atmospheric VOCs during the Continuous Haze and Non-Haze Episodes in the Urban Area of Beijing: A Case Study in Spring of 2019. *Atmosphere* **2021**, *12*, doi:10.3390/atmos12020171.
5. Yao, D.; Tang, G.; Wang, Y.; Yang, Y.; Wang, L.; Chen, T.; He, H.; Wang, Y. Significant contribution of spring northwest transport to volatile organic compounds in Beijing. *J. Environ. Sci.* **2021**, *104*, 169-181, doi:10.1016/j.jes.2020.11.023.
6. Liu, Y.; Song, M.; Liu, X.; Zhang, Y.; Hui, L.; Kong, L.; Zhang, Y.; Zhang, C.; Qu, Y.; An, J.; et al. Characterization and sources of volatile organic compounds (VOCs) and their related changes during ozone pollution days in 2016 in Beijing, China. *Environ Pollut* **2020**, *257*, 113599, doi:10.1016/j.envpol.2019.113599.
7. Luo, D.T.; Zhang, Q.M.; Liu, Z.; You, X.Y.; Huang, J.; Song, Y.F.; Zhang, J.Q. Characteristics and Source Apportionment of Volatile Organic Compounds in August in the Chang-Zhu-Tan Urban Area. *Huan Jing Ke Xue* **2022**, *43*, 3463-3472, doi:10.13227/j.hjkx.202109163.
8. Xiong, C.; Wang, N.; Zhou, L.; Yang, F.; Qiu, Y.; Chen, J.; Han, L.; Li, J. Component characteristics and source apportionment of volatile organic compounds during summer and winter in downtown Chengdu, southwest China. *Atmos. Environ.* **2021**, *258*, doi:10.1016/j.atmosenv.2021.118485.
9. Tan, Q.; Liu, H.; Xie, S.; Zhou, L.; Song, T.; Shi, G.; Jiang, W.; Yang, F.; Wei, F. Temporal and spatial distribution characteristics and source origins of volatile organic compounds in a megacity of Sichuan Basin, China. *Environ. Res.* **2020**, *185*, doi:10.1016/j.envres.2020.109478.
10. Li, J.; Zhai, C.; Yu, J.; Liu, R.; Li, Y.; Zeng, L.; Xie, S. Spatiotemporal variations of ambient volatile organic compounds and their sources in Chongqing, a mountainous megacity in China. *Sci. Total Environ.* **2018**, *627*, 1442-1452, doi:10.1016/j.scitotenv.2018.02.010.
11. Zhou, Z.; Xiao, L.H.; Fei, L.L.; Yu, W.; Lin, M.; Huang, J.J.; Zhang, Z.S.; Tao, J. Characteristics and Source Apportionment of Volatile Organic Compounds (VOCs) in a Typical Industrial Area in Dongguan During Periods of Ozone and Non-ozone Pollution in Summer. *Huan Jing Ke Xue* **2022**, *43*, 4497-4505, doi:10.13227/j.hjkx.202111285.
12. He, Z.; Wang, X.; Ling, Z.; Zhao, J.; Guo, H.; Shao, M.; Wang, Z. Contributions of different anthropogenic volatile organic compound sources to ozone formation at a receptor site in the Pearl River Delta region and its policy implications. *Atmos. Chem. Phys.* **2019**, *19*, 8801-8816, doi:10.5194/acp-19-8801-2019.
13. Meng, Y.; Song, J.; Zeng, L.; Zhang, Y.; Zhao, Y.; Liu, X.; Guo, H.; Zhong, L.; Ou, Y.; Zhou, Y.; et al. Ambient volatile organic compounds at a receptor site in the Pearl River Delta region: Variations, source apportionment and effects on ozone formation. *J. Environ. Sci.* **2022**, *111*, 104-117, doi:10.1016/j.jes.2021.02.024.
14. Chen, C.; Wang, L.; Zhang, Y.; Zheng, S.; Tang, L. Spatial and Temporal Distribution Characteristics and Source Apportionment of VOCs in Lianyungang City in 2018. *Atmosphere* **2021**, *12*, doi:10.3390/atmos12121598.
15. Liu, Y.; Wang, H.; Jing, S.; Gao, Y.; Peng, Y.; Lou, S.; Cheng, T.; Tao, S.; Li, L.; Li, Y.; et al. Characteristics and sources of volatile organic compounds (VOCs) in Shanghai during summer: Implications of regional transport. *Atmos. Environ.* **2019**, *215*, doi:10.1016/j.atmosenv.2019.116902.
16. Hui, L.; Liu, X.; Tan, Q.; Feng, M.; An, J.; Qu, Y.; Zhang, Y.; Jiang, M. Characteristics, source apportionment and contribution of VOCs to ozone formation in Wuhan, Central China. *Atmos. Environ.* **2018**, *192*, 55-71, doi:10.1016/j.atmosenv.2018.08.042.
17. Hui, L.R.; Liu, X.G.; Tan, Q.W.; Feng, M.; An, J.L.; Qu, Y.; Zhang, Y.H.; Cheng, N.L. VOC characteristics, sources and contributions to SOA formation during haze events in Wuhan, Central China. *Sci. Total Environ.* **2019**, *650*, 2624-2639.
